# AUX/LAX family of auxin influx carriers—an overview

**DOI:** 10.3389/fpls.2012.00225

**Published:** 2012-10-18

**Authors:** Ranjan Swarup, Benjamin Péret

**Affiliations:** ^1^School of Biosciences and Centre for Plant Integrative Biology, University of NottinghamLoughborough, UK; ^2^Laboratory of Plant Development Biology, SBVME/Institute for Biotechnology and Environmental Biology, CEA CadaracheSt. Paul lez Durance, France

**Keywords:** AUXLAX, auxin transport, auxin, AUX1, LAX1, LAX2, LAX3, influx carriers

## Abstract

Auxin regulates several aspects of plant growth and development. Auxin is unique among plant hormones for exhibiting polar transport. Indole-3-acetic acid (IAA), the major form of auxin in higher plants, is a weak acid and its intercellular movement is facilitated by auxin influx and efflux carriers. Polarity of auxin movement is provided by asymmetric localization of auxin carriers (mainly PIN efflux carriers). PIN-FORMED (PIN) and P-GLYCOPROTEIN (PGP) family of proteins are major auxin efflux carriers whereas AUXIN1/LIKE-AUX1 (AUX/LAX) are major auxin influx carriers. Genetic and biochemical evidence show that each member of the AUX/LAX family is a functional auxin influx carrier and mediate auxin related developmental programmes in different organs and tissues. Of the four *AUX/LAX* genes, AUX1 regulates root gravitropism, root hair development and leaf phyllotaxy whereas LAX2 regulates vascular development in cotyledons. Both AUX1 and LAX3 have been implicated in lateral root (LR) development as well as apical hook formation whereas both AUX1 and LAX1 and possibly LAX2 are required for leaf phyllotactic patterning.

## Introduction

Genetic, molecular and pharmacological approaches have elegantly demonstrated that auxin regulates several aspects of plant growth and development including embryo (Steinmann et al., 1999; Wolters et al., 2011), root (Swarup et al., 2001, 2004, 2005), lateral root (LR) (Swarup et al., 2008; Péret et al., 2009a,b), leaf (Bainbridge et al., 2008; Guenot et al., 2012) and flower development. Auxin also plays a key role in plant tropic responses (Swarup et al., 2001, 2004, 2005), vascular development (Sieburth and Deyholos, 2006; Péret et al., 2012) and regulation of apical dominance (Aloni et al., 2006; Prusinkiewicz et al., 2009). At cellular level, auxin regulates cell division, cell elongation and cell differentiation (Petrásek and Friml, 2009; Vanneste and Friml, 2009).

Indole-3-acetic acid (IAA) is the major form of auxin in higher plants and was the first plant hormone to be discovered (Went, 1926). Besides, there are a few other naturally occurring auxins. Auxins are organic compounds composed of an indole ring covalently linked to a carboxylic acid group (or a benzene ring in the case of phenylacetic acid—PAA). In addition, several synthetic compounds with auxin like activities have also been identified. Of them 2,4-dichlorophenoxyacetic acid (2,4-D) is one of the most widely used in auxin research.

Auxin is unique among all plant hormones for exhibiting polar transport. It is primarily synthesized in the shoot apex and developing leaf primordia and is then transported either through the bulk flow in the phloem in a non-polar fashion or actively in a polar manner to distal target tissues (Swarup and Bennett, 2003).

## Auxin distribution: simply complex

Use of auxin response reporters for example DR5 (Ulmasov et al., 1997) and IAA2 (Abel et al., 1994) and auxin sensors DII 28 (Brunoud et al., 2012) have provided great insight into auxin accumulation and distribution in plant tissues. These studies show that auxin gradients are crucial for several aspects of plant development including tropic responses, organ development and meristem size. For example, several studies show that differential accumulation of auxin between lower and upper side of a gravistimulated root regulate root bending (Ottenschläger et al., 2003; Swarup et al., 2005); auxin maxima are known to regulate organ development (Sabatini et al., 1999; Benková et al., 2003; Blilou et al., 2005; Grieneisen et al., 2007) and even auxin minimum has been implicated in regulating seed dispersal in *Arabidopsis* (Sorefan et al., 2009). Genetic and pharmacological studies show that auxin transport is crucial for establishment of auxin gradients and disruption of these gradients result in several auxin related developmental defects. Besides auxin transport, local auxin biosynthesis, metabolism, conjugation/deconjugation of active auxins to/from their inactive conjugated forms and intracellular auxin movement can also control and fine tune auxin accumulation in specific cell or tissues types (Chandler, 2009; Ikeda et al., 2009; Petrásek and Friml, 2009; Vanneste and Friml, 2009).

## Auxin transporters: providing direction

**Figure 1:**
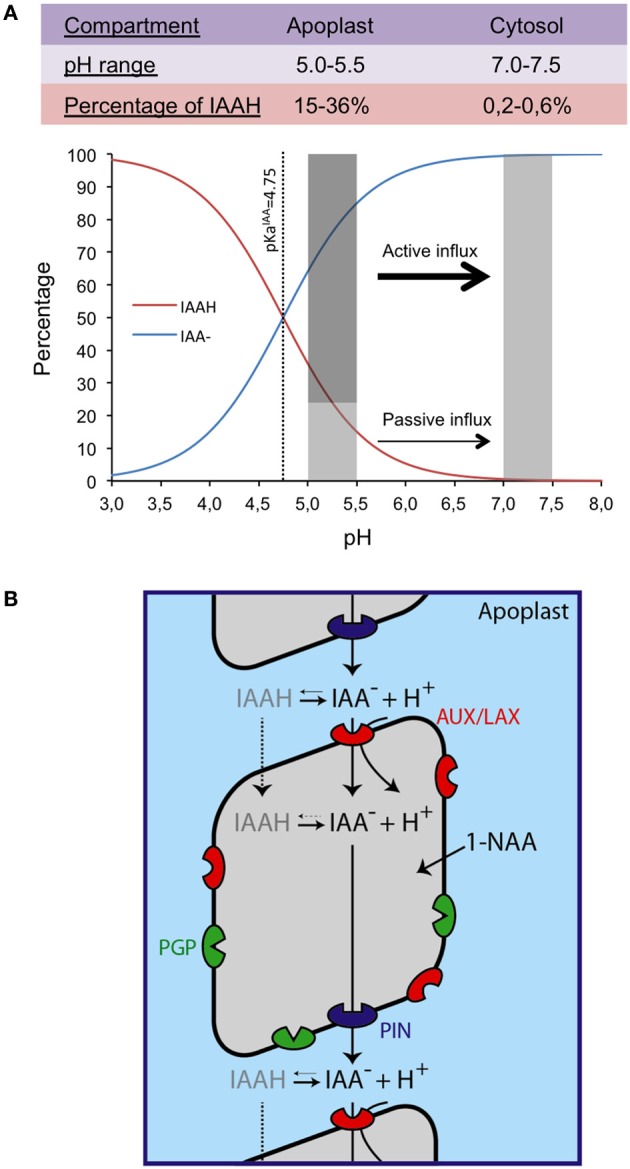
**Auxin chemical properties and chemioosmotic hypothesis of auxin transport.** The percentages of the anionic and protonated forms of IAA (indole acetic acid) are given as a funtion of pH with an emphasis on the apoplastic and cytosolic pH ranges **(A)**. Chemiosmotic auxin transport model showing the different families of transporters: AUX/LAX, PIN, and PGP. The artificial form of auxin 1-NAA (1-Naphthalene acetic acid) is lipophilic and diffuses freely inside the cell **(B)**.

As per chemiosmotic polar diffusion hypothesis, the term first coined by Goldsmith (1977) based on the famous work of Rubery and Sheldrake (1974) and Raven (1975) cellular IAA movement is facilitated by combined activities of auxin influx and efflux carriers. IAA is a weak acid (pKa 4.75) and at mildly acidic apoplastic pH, only a small portion of IAA (IAAH ~15%) is able to passively diffuse inside the cell but the majority (85%) of IAA remains in its dissociated form (IAA^−^) and would require a carrier for its active uptake across the cell (Figure 1A). Inside the cell, at pH 7.0, all IAA remains in its polar IAA^−^ form and would require auxin efflux carriers (Zazímalová et al., 2010). Chemiosmotic hypothesis also predicted that the polarity of auxin movement is provided by asymmetric localization of auxin carriers.

In *Arabidopsis*, evidence has been provided that AUXIN1/LIKE-AUX1 (AUX/LAX) family of auxin transporters are major influx carriers whereas PIN-FORMED (PIN) and P-GLYCOPROTEIN (PGP) family members are major auxin efflux carriers (Figure 1B). Among the efflux carriers, PIN family is most well studied and PIN homologs are found throughout the plant kingdom (Paponov et al., 2005; Pattison and Catalá, 2011; Wang et al., 2011; Carraro et al., 2012). In *Arabidopsis*, PINs are encoded by a small gene family comprising of eight members (Grunewald and Friml, 2010; Bosco et al., 2012). They have been shown to play crucial roles in several aspects of plant growth and development including root meristem patterning, LR development, vascular development and embryo development (Friml et al., 2002; Benková et al., 2003; Friml et al., 2003; Reinhardt, 2003; Blilou et al., 2005; Sieburth and Deyholos, 2006). PIN proteins are localized either on the plasma membrane (PIN1, 2, 3, 4, and 7) or in the ER (PIN5 and 8) and thus play a key part in both intercellular and intracellular auxin movement and regulation of auxin homeostasis (Mravec et al., 2009; Bosco et al., 2012). It is now well established that directionality of auxin movement is provided by asymmetric localization of PIN proteins. For example, PIN1 is localized on the basal rootward face of vascular cells (Gälweiler et al., 1998) facilitating rootward movement of auxin. In contrast, PIN2 is asymmetrically localized at the apical shootward face of LRC and epidermal cells and basal rootward face of cortical cells of the meristem thus creating an auxin reflux loop (Blilou et al., 2005; Wisniewska et al., 2006; Rahman et al., 2010). In response to gravity PIN3 is asymmetrically localized on the lateral face of the root to facilitate differential movement of auxin between upper and lower faces of a gravi-stimulated root (Friml et al., 2002).

In addition to PINs, a novel PIN like family of auxin transport facilitators termed PILS (PIN-LIKES) has recently been discovered by *in silico* studies and appears to be involved in the regulation of auxin homeostasis in *Arabidopsis* (Barbez et al., 2012).

Three members of the PGP class of ABC transporters PGP1, PGP4, and PGP19 have also been implicated in regulating auxin transport. Both PGP1 and PGP19 are involved in auxin efflux (Noh et al., 2003; Blakeslee et al., 2007), PGP4 has been demonstrated to participate in the shootward (basipetal) redirection of auxin from the root apex and there is some evidence to suggest that PGP4 functions as an auxin influx carrier (Terasaka et al., 2005; Kubeš et al., 2012). However, Cho et al. (2007) showed that PGP4 functions as an auxin efflux carrier. Direct auxin measurement experiment in heterologous expression system suggests that PGP4 can indeed function both as an efflux and influx carrier (Yang and Murphy, 2009).

Recently, a role for the nitrate transporter NRT1.1 in auxin influx has been demonstrated in heterologous system, providing an explanation for its ability to alter LR formation depending on the nitrogen status of the plant (Krouk et al., 2010). Interestingly, NRT1.1 acts as a transceptor as it is also involved in the perception/transduction of the nitrate signal (Ho et al., 2009). Further understanding of the auxin transport function of NRT1.1 is of great interest as this provides a direct mechanism for developmental effects of auxin in response to nutrient status of the soil.

## The AUX/LAX family of auxin influx carriers: historical perspective

The existence of auxin influx carriers was first suggested by (Rubery and Sheldrake, 1974) when they showed a saturable component for auxin uptake in *Parthenocissus tricuspidata* crown gall suspension cells. Using sealed zucchini membrane vesicles, Lomax et al. (1985) provided further evidence that IAA uptake is an active process and is driven by proton motive force. They also proposed that auxin influx carrier acts as a proton symporter that was later confirmed by Sabater and Rubery (1987). In 1996, Delbarre et al. showed that the synthetic auxin 2, 4-D was a substrate for auxin influx carrier but not the lipophilic auxin 1-naphthalene acetic acid (1-NAA) that is able to diffuse freely into the cells (Figure 1B). They also showed that almost 75% of 2,4-D uptake was carrier mediated thus underlining the importance of auxin influx carriers in auxin uptake.

In the same year Bennett et al. (1996) cloned the *AUX1* gene. *aux1* mutants are agravitropic and were first identified in an screen for auxin (2,4-D) resistance (Maher and Martindale, 1980). *AUX1* gene showed similarity to amino acid transporters and the fact that IAA is structurally similar to tryptophan led Bennett et al. to propose that *AUX1* encodes a putative auxin influx permease. Detailed characterization of *aux1* mutants revealed that they show selective resistance to various auxins and root agravitropic defect of *aux1* can be rescued by application of lipophilic auxin 1-NAA (Yamamoto and Yamamoto, 1998; Marchant et al., 1999). Swarup et al. (2001) later showed that in *Arabidopsis* roots, besides in protophloem, *AUX1* is expressed in tissues that are involved in gravity perception (columella), signal transmission (LRC) and response (epidermis). They also were able to provide a molecular basis of *aux1* root gravitropic phenotype when they showed that *aux1* mutants were defective in basipetal auxin transport. The first direct evidence to show that AUX1 is an auxin permease came from Erik Nielsen's group when they expressed AUX1 in *Xenopus laevis* oocytes and showed a saturable, pH dependent increase in IAA uptake (Yang et al., 2006). Their experiments provided the first direct evidence that AUX1 is a high affinity auxin transporter. Later Carrier et al. (2008) provided first direct evidence of the affinity of an auxin influx carrier for its cognate ligand. They provided evidence that IAA binds to AUX1 in a pH dependent fashion with maximal binding taking place between pH5 and 6.

## The AUX/LAX family: a case for subfunctionalization

AUX/LAX homologs have been reported to be present throughout the plant kingdom (Hochholdinger et al., 2000; de Billy et al., 2001; Kamada et al., 2003; Schrader et al., 2003; Schnabel and Frugoli, 2004; Péret et al., 2007; Hoyerová et al., 2008; Oliveros-Valenzuela et al., 2008; Shen et al., 2010; Pattison and Catalá, 2011; Carraro et al., 2012) and may have evolved before the evolution of land plants as *AUX/LAX* like sequences have been reported to be present in several single-celled and colony-forming Chlorophyta species (De Smet et al., 2011).

**Figure 2:**
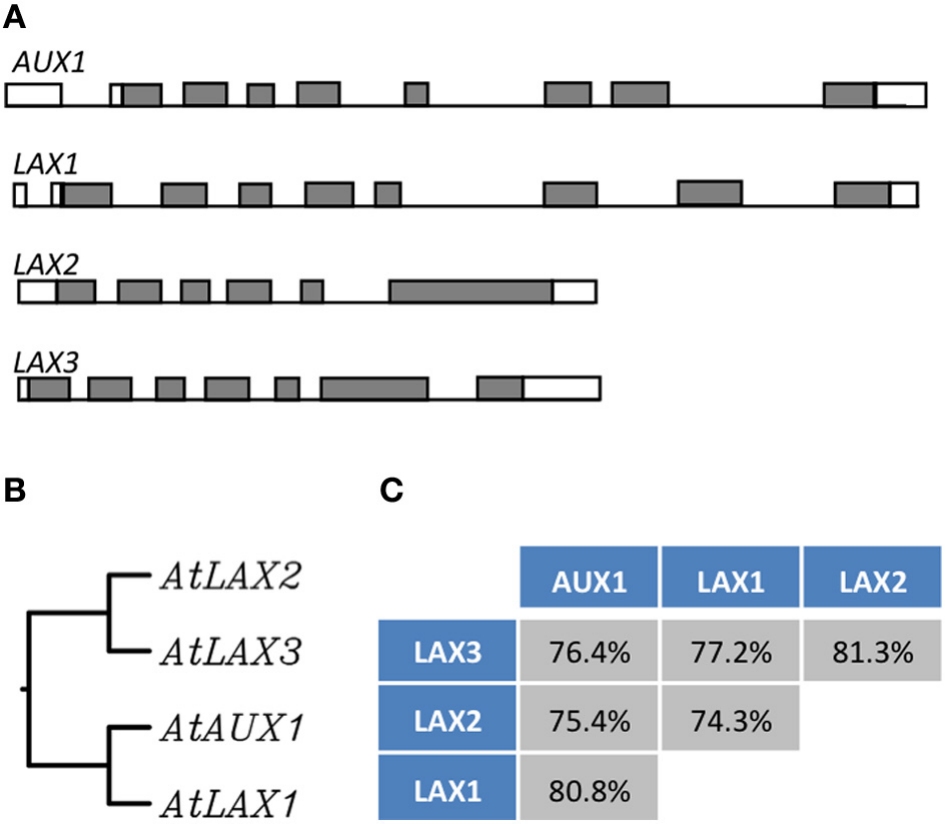
**The AUX/LAX family of auxin influx transporters.** Genetic organization of the *AUX/LAX* genes sequences showing exons (gray boxes) and introns (bars) (Péret et al., 2012) **(A).** Phylogenetic tree of the AUX/LAX protein sequences generated from a ClustalW alignment **(B)**. Percentage of identity between the members of the AUX/LAX family (identity is given a the score returned upon the ClustalW alignment) **(C)**.

**Figure 3:**
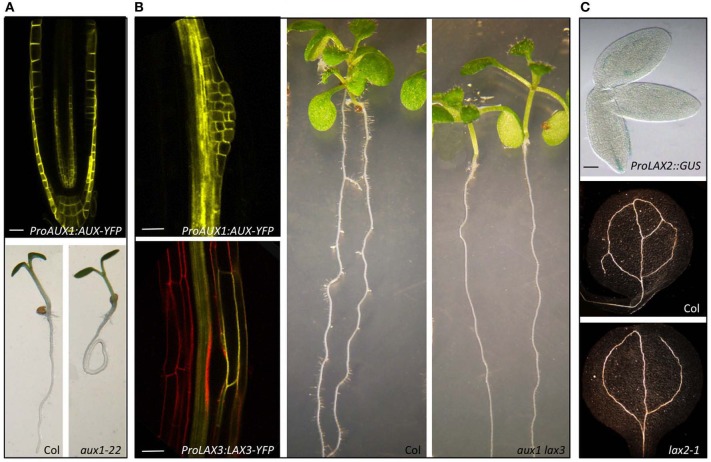
**Mutations in *AUX/LAX* genes result in auxin related developmental defects.** AUX1 regulates root gravitropism (Swarup et al., 2001, 2004, 2005). *AUX1* is expressed in tissues that are involved in gravity perception, signal transmission, and response and mutation in *aux1* cause agravitropic roots **(A)**. Both AUX1 and LAX3 regulate lateral root development (Swarup et al., 2008). *AUX1* is expressed in lateral root primordia whereas *LAX3* in the cortical and epidermal cells in contact with the primordia and *aux1 lax3* double mutants have severely delayed lateral root emergence **(B)**. LAX2 regulates vascular patterning in cotyloedons (Péret et al., 2012). *LAX2* is expressed in the vascular tissues during embryo development and *lax2* mutants show vascular breaks in the cotyledons **(C)**. (Scale bars 20 μm).

In *Arabidopsis*, *AUX1* belongs to a small gene family comprising of four highly conserved genes, *AUX1* and *LIKE-AUX1* (*LAX*) genes, *LAX1*, *LAX2*, and *LAX3* and form a plant- specific subclass within the amino acid/auxin permease (AAAP) super family (Young et al., 1999; Péret et al., 2012) (Figure 2). These genes encode multi membrane spanning transmembrane proteins. In a very elegant study, Swarup et al. (2004) using a pH sensitive YFP as a probe to determine the topology of AUX1 showed that AUX1 has 11 transmembrane segments with N terminal residing inside the cell and C-terminal outside. *AUX/LAX* genes share extensive sequence similarity (Péret et al., 2012). There is ample evidence to suggest that these genes have originated from a common ancestor through gene duplication. For example, AUX1 shares 82, 78, and 76% identity with LAX1, LAX2, and LAX3, respectively, and they also show well conserved gene structure (Péret et al., 2012) (Figure 2), At functional level evidence has been provided that these genes encode functional auxin influx carriers (Yang et al., 2006; Swarup et al., 2008; Péret et al., 2012) and mutations in these genes result in auxin related developmental defects (Figure 3; Bennett et al., 1996; Swarup et al., 2001, 2004, 2005, 2007, 2008; Bainbridge et al., 2008; Péret et al., 2012). Despite the conservation of biochemical function, these genes show mostly non-redundant expression and during the course of evolution have subfunctionalized to facilitate auxin related developmental programmes in different plant organs and tissues as reviewed below.

## Root gravitropism: AUX1 at the helm

The founder member of the AUX/LAX family, AUX1 is well documented to play a key role in root gravitropic response. *AUX1* is expressed in tissues that are involved in gravity perception, signal transmission and response (Figure 3A). Mutation in *AUX1* results in severely agravitropic roots. Using an auxin responsive *IAA2:GUS* reporter, Swarup et al. (2001) showed that *aux1* mutants had defects in auxin movement from the root apex to the distal elongation zone. Later using a transactivation based approach, Swarup et al. (2005) mapped the auxin transport route during a gravitropic response and provided evidence that AUX1 was important for facilitating movement of auxin from the site of gravi-perception to gravi-response. Computer simulations of auxin fluxes through elongation zone tissues suggest that expression of auxin influx carrier *AUX1* and efflux carrier *PIN2* in the epidermis minimize the effect of radial diffusion while facilitating basipetal auxin transport (Swarup et al., 2005). Thus while PIN2 provides directionality of auxin movement, AUX1 appears essential for the efficient auxin uptake by expanding epidermal cells. More recently, Monshausen et al. (2011) have provided further insight into the importance of AUX1 in root gravitropism. Using confocal microscopy and fluorescent pH sensors, they show that there is an increase in the surface pH on the lower side of a gravistimulated wildtype but not *aux1* roots. One important implication of this finding is that increase in the root apoplastic pH will result in more IAA in its ionic IAA^−^ form. IAA^−^ is not membrane permeable and will require a carrier (AUX1) mediated uptake. This work helps to clarify a common misconception that because protonated IAA is membrane permeable, influx carriers play only a supplemental role and backs up computer simulation studies that estimate that carrier mediated IAA uptake is 15 times greater than the diffusion when *AUX1* is expressed in the root epidermal cells (Swarup et al., 2005; Kramer and Bennett, 2006).

Except AUX1, no other member of the AUX/LAX family plays a role in root gravitropic response (Péret et al., 2012). Apart from some expression of *LAX2* and *LAX3* in the columella cells, none of them are expressed in the tissues that are involved in gravity signal transmission (LRC) or response (epidermis). Also both *lax2* and *lax3* single mutants do not show any root gravitropic defect and *lax2 aux1* double mutants are no more severe than *aux1* (Péret et al., 2012).

## Lateral root development: the emerging story

**Figure 4:**
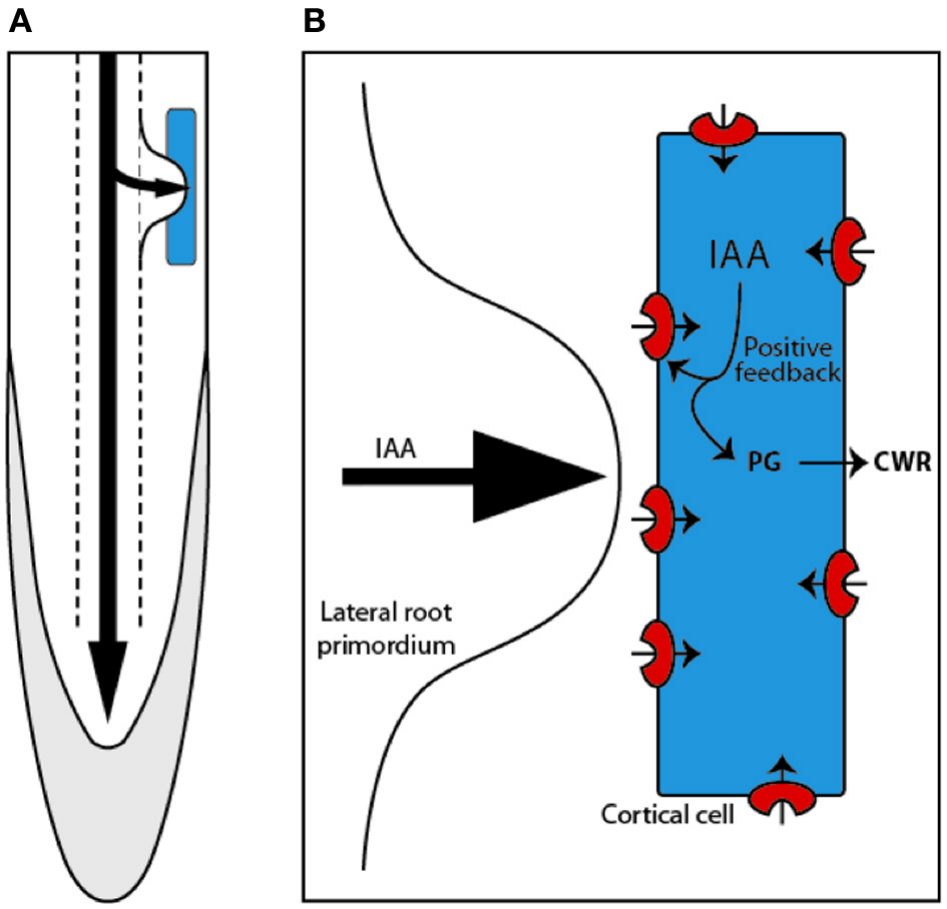
**Lateral root are formed within the pericycle deep inside the primary root and have to emerge through the outer tissue, passing through the endodermal, cortical (blue), and epidermal cells (A)**. Mechanism proposed by Swarup et al. (2008) describing how auxin (IAA) entering the cortical cell induces the expression of *LAX3*. This generates the establishment of a positive feedback loop that triggers high auxin levels and subsequent induction of cell wall remodeling (CWR) genes, such as the *polygalacturonase (PG)* **(B)**.

LRs originate from the pericycle cells that divide and self organize to create a new primordium (Dubrovsky et al., 2000, 2001). As the LR formation process occurs deep inside the primary root tissues (Figure 4A), the newly formed organ has to penetrate through several layers of cells ranging from 3 in *Arabidopsis* (Swarup et al., 2008; Péret et al., 2009a,b) to as many as 15 in rice (Rebouillat et al., 2008). Several lines of evidences implicate auxin in LR initiation and development (Péret et al., 2009a,b).

The initiation phase starts when two adjacent pericycle cells start to divide asymmetrically and create a LR primordium (Péret et al., 2009a,b). This process is associated with the creation of an auxin maximum in the pericycle founder cells (Benková et al., 2003; De Smet et al., 2007). Auxin influx carriers have been implicated in regulating LR development (Marchant et al., 2002; De Smet et al., 2007; Swarup et al., 2008) Marchant et al. (2002) demonstrated that *AUX1* is expressed in the pericycle cells before the first periclinal division and the *aux1* mutant displays a 50% reduction in the number of LRs (Hobbie and Estelle, 1995). Analysis of the auxin response reporter *IAA2:GUS* revealed that auxin content and distribution is altered in the *aux1* mutant that led Marchant et al. (2002) to conclude that AUX1 facilitates IAA loading into the vascular transport system.

Working on *LAX3*, Swarup et al. (2008) provided evidence that auxin influx carriers also regulate LR emergence (Swarup et al., 2008). They discovered that mutations in auxin influx carrier *LAX3* resulted in reduced number of emerged LR. Interestingly they found that the total number of initiation events was increased in *lax3* and this led them to suggest that initiation and emergence compete for the same source of auxin (Lucas et al., 2008a,b).

Molecular characterization of *LAX3* by Swarup et al. (2008) revealed that *LAX3* is expressed in the cortical and epidermal cells specifically situated in front of the LR primordia (Figure 3B). From Benková et al. (2003) work, they knew that auxin maxima is localized in the LR primordia and this led them to test the tantalising possibility that auxin itself could be the signal for *LAX3* expression in front of the primordia. Indeed, *LAX3* turned out to be auxin inducible. But how does LAX3 facilitate emergence? To find answer, Swarup et al. (2008) used a substractive transcriptomics approach to identify genes that are co expressed with *LAX3* in outer tissues and discovered that several cell wall remodeling genes were expressed in these cells in *LAX3* dependent fashion. Progression of the primordium inside the root tissues has long been associated with production of cell wall remodeling enzymes (Cosgrove, 2000, 2005) and this led Swarup et al. (2008) to propose that auxin from the LR primordia enters the cortical cells and induces *LAX3* expression (Figure 4). The activity of LAX3 at the plasma membrane is then proposed to facilitate auxin uptake in the same cell and would reinforce *LAX3* expression. As a result more and more auxin would accumulate in the cortical cells that will result in the induction of cell wall remodeling enzymes that is then proposed to facilitat smooth passage of the primordium through the cortex. The similar mechanism can then allow primordia passage through the epidermis. Therefore, as per this hypothesis, LAX3 participates in the creation of an auxin sink in a few cells and its expression in the outer tissues is dependent on its position compared to the source of auxin (the LR primordium) resulting in a typical “all or nothing” response.

## Root hair development: back seat driving

As the roots grow, old cells are continuously being pushed upwards and they pass through zones of elongation and differentiation. Root hairs are produced from a subset of epidermal cells in the differentiation zone. Auxin plays a key role in several aspects of root development including maintenance of the root apical meristem (Blilou et al., 2005); epidermal cell development (Sabatini et al., 1999; Grieneisen et al., 2007) and initiation and continued growth of root hairs (Pitts et al., 1998; Grebe et al., 2002; Rahman et al., 2002; Knox et al., 2003; Fischer et al., 2006). Interestingly, despite the importance of auxin in root hair development, no auxin influx carrier is expressed in the root hair cells. Jones et al. (2009) discovered that *AUX1* is expressed in the neighboring non-hair cells. In contrast to *AUX1*, auxin efflux carrier *PIN2* is expressed in both hair and non-root hair cells. Despite no *AUX1* being expressed in the hair cells, root hair length in the *aux1* mutant was shorter but can be restored to wildtype levels by treatment with exogenous auxin clearly implicating AUX1 in root hair growth. Furthermore, epidermal expression of *AUX1* was not detected in *werewolf/myb23* mutants that lack non-hair cells. These mutants have shorter root hairs but can be restored to wildtype levels by auxin treatment. This led Jones et al. (2009) to conclude that non-hair cells affect auxin abundance in hair cells. Computer simulation studies indicate that expression of *AUX1* in the non-hair cells result in over 10 fold accumulation of auxin in these cells compared to the adjacent hair cells. Due to the PIN2 activity, auxin can be effluxed out of the non-hair cells and into the apoplast and despite the lack of AUX1 in the hair cells, high auxin concentration can still be maintained in the hair cells in the differentiation zone up to 500 μm from the root apex. In contrast, in the *aux1* mutants, there will be significantly less accumulation of auxin in the root hair cells as due to the slow rate of diffusion, most of the auxin will either be recycled to the vascular tissues or will be lost through the epidermis before it reaches the differentiation zone (Jones et al., 2009). Thus their work suggests that AUX1 helps to maintain high auxin levels in the differentiation zone and facilitates root hair growth.

AUX1 has also been implicated in maintenance of hair cell polarity (Grebe et al., 2002). Root hairs are formed on the basal side of the hair cells but they initiate from a more basal position in presence of auxin. Mutation in *aux1* results in apical shifting of the root hairs. *aux1* mutants also had 30 times higher frequency of double hair formation compared to wildtype. These results provided a clear link between auxin transport and the establishment of apical-basal epidermal polarity in *Arabidopsis*.

## Embryonic root cell organization: size matters

*Arabidopsis* root meristem is highly organized and a combination of apical basal and radial patterning inputs establish the positioning of the stem cell niche (Scheres, 2007). Both genetic and pharmacological approaches show that auxin transport plays a key role in this process. Working in *Arabidopsis* embryo, Ugartechea-Chirino et al. (2010) provided first evidence for the role of auxin influx carriers in patterning of the embryonic root. They showed that the quadruple *aux/lax* mutants had severely disorganized radicle apex and had significant increase in the root-cap cell number, average cell size, or both.

## Vascular development: a role for LAX2

Genetic and pharmacological studies have clearly shown that auxin regulates vascular development (Reinhardt, 2003; Petrásek and Friml, 2009). Recently, Péret et al. (2012) provided evidence that LAX2 is important for vascular development in cotyledons (Figure 3C). Using a *promoter:GUS* approach they show that *LAX2* is expressed in procambial and vascular tissues during embryogenesis. Examination of the *lax2* mutants revealed that they had higher propensity of discontinuity in vascular strands in the cotyledons. Though *LAX2* expression is also detected very early in developing leaves at the sites of initiating veins surprisingly, Péret et al. (2012) did not find any apparent defect in vascular patterning in *lax2* leaves. *AUX1* is also expressed in developing leaves and it will be interesting to see if *lax2 aux1* and quadruple *aux/lax* mutants show any defect in vascular patterning in leaves.

## Apical hook development: cross talk at its best

In dicotyledonous seedlings, apical hook protects the meristem when seedlings emerge from the soil. In the light, apical hooks opens, cotyledons expand and the photosynthesis begins (Chen and Chory, 2011). Besides light, plant hormones auxin, ethylene, gibberellins, and brassinosteroids are crucial for the maintenance and development of apical hook. Using a very elegant transactivation based approach Vandenbussche et al. (2010) showed that the auxin response maximum on the concave side is essential for correct hook development. The first evidence to implicate auxin influx carriers in apical hook development was provided by Roman et al. (1995) when they showed that *aux1* mutants are defective in hook development, a finding later confirmed by Stepanova et al. (2007). More recently, Vandenbussche et al. (2010) have provided evidence that LAX3 is also involved in apical hook development. Using single and multiple *aux/lax* mutant combinations, they showed that *lax3* mutants had partial hookless phenotype. They also showed that upon treatment with ethylene, both *aux1* and *lax3* had less exaggerated apical hook and *aux1 lax3* double mutants apical hook defect was as severe as seen when treated with auxin influx inhibitor 1-NOA (1-naphthoxyacetic acid). More detailed characterization including kinetics of hook development led Vandenbussche et al. (2010) to conclude that LAX3 is the major auxin influx carrier in hook development assisted by AUX1 and AUX1 appears to play a major role in ethylene-mediated hook exaggeration.

## Phyllotactic patterning: team work

Phyllotaxy is the arrangement of organ primordia on a plant stem. Spiral phyllotaxy is the most common phyllotactic patterns in nature where new organ primorida initiates roughly at an angle of 137.5° and has intrigued biologists for generations (Fleming, 2005). Auxin transport appears to be crucial for the development of phyllotactic patterns. Auxin response reporter DR5 based studies in *Arabidopsis* show that auxin maxima is localized at the site where new primordia originate (Benková et al., 2003; Heisler et al., 2005; Smith et al., 2006). Reinhardt et al. (2003) showed that asymmetric localization of auxin efflux carrier PIN1 is important for the establishment of this auxin maxima that provides instructive signal for the formation of primordia. Stieger et al. (2002) provided evidence for a role for auxin influx carriers in phylltactic patterning. Using inhibitors of auxin influx carrier (Parry et al., 2001; Lankova et al., 2010), they revealed that auxin influx carriers were required for proper localization of leaf primordia. Further proof for the involvement of auxin influx carriers in phyllotactic patterning was provided by Reinhardt et al. (2003) and Bainbridge et al. (2008). Working on *pin1* mutants, Reinhardt et al. (2003) showed that localized auxin application on *pin1* meristem can restore primordia formation but such localized auxin application on *aux1 pin1* double mutants resulted in wider primordia formation. Using single and multiple *aux lax* mutants and their combinations Bainbridge et al. (2008) then showed that *AUX/LAX* genes act redundantly to regulate phyllotactic patterning in *Arabidopsis*. They revealed that in the *aux/lax* quadruple mutant, primordia formed at irregular angles (as compared to 137.5° in controls) and unusually often showed primordia clusters. Study of the multiple *aux/lax* mutant combinations revealed that besides quadruple, *aux1 lax1 lax2*, *aux1 lax1 lax3*, and *aux1 lax1* combinations had defect in phyllotactic patterning. They also reported that patterning in inflorescence meristem was also defective in the same mutant combinations. This led them to conclude that AUX1 and LAX1 act redundantly to regulate phyllotactic patterning in *Arabidopsis*. They also discovered that the phyllotactic defect in quadruple and *aux1 lax1 lax2* mutants was more severe compared to *aux1 lax1 lax3* and *aux1 lax1* mutants combination. On this basis they conclude that LAX2 may have a redundant function in regulating phyllotactic patterning. Interestingly *LAX2* is expressed in the vasculature but not expressed in the shoot apical meristem itself and to account for its involvement in phyllotactic patterning, Bainbridge et al. (2008) propose that LAX2 may increase the sink strength by pulling auxin out of the L1 layer and thus inhibiting primordium formation in this region.

## Role of AUX-LAX genes in biotic interactions

Many plant-associated bacteria are known to synthesize auxins, including IAA, which leads to diverse outcomes for the plant ranging from simple growth stimulation to promoting symbiotic interactions and even pathogenesis (Spaepen et al., 2007). Sequencing of several bacterial genomes has revealed the existence of different auxin synthesis pathways with a high degree of similarity with plant pathways (Spaepen et al., 2007). For example, in actinobacterium *Frankia*, at least two auxin synthesis pathways have been identified correlating with the production of two naturally occurring auxins: IAA and PAA. Interestingly, production of both these auxins is increased in nitrogen-deprived medium (Perrine-Walker et al., 2010). Furthermore, nitrogen deprivation promotes nitrogen-fixing symbiosis demonstrating that the establishment of this symbiotic interaction can be modulated by environmental (and genetic) factors. On the other hand, manipulation of auxin perception in the plant hosts appears to be a common mechanism during plant-microbe interactions. For instance, a plant miRNA induced by *Pseudomonas syringae* flagellin-derived peptide reduces the expression of the auxin receptor *TIR1* and its homologs *AFB2* and *AFB3* (Navarro et al., 2006). In recent years evidence is emerging that auxin transport may also play a key role in both symbiotic and pathogenic plant microbe interactions affecting penetration of auxin in the host plant cell.

**Figure 5:**
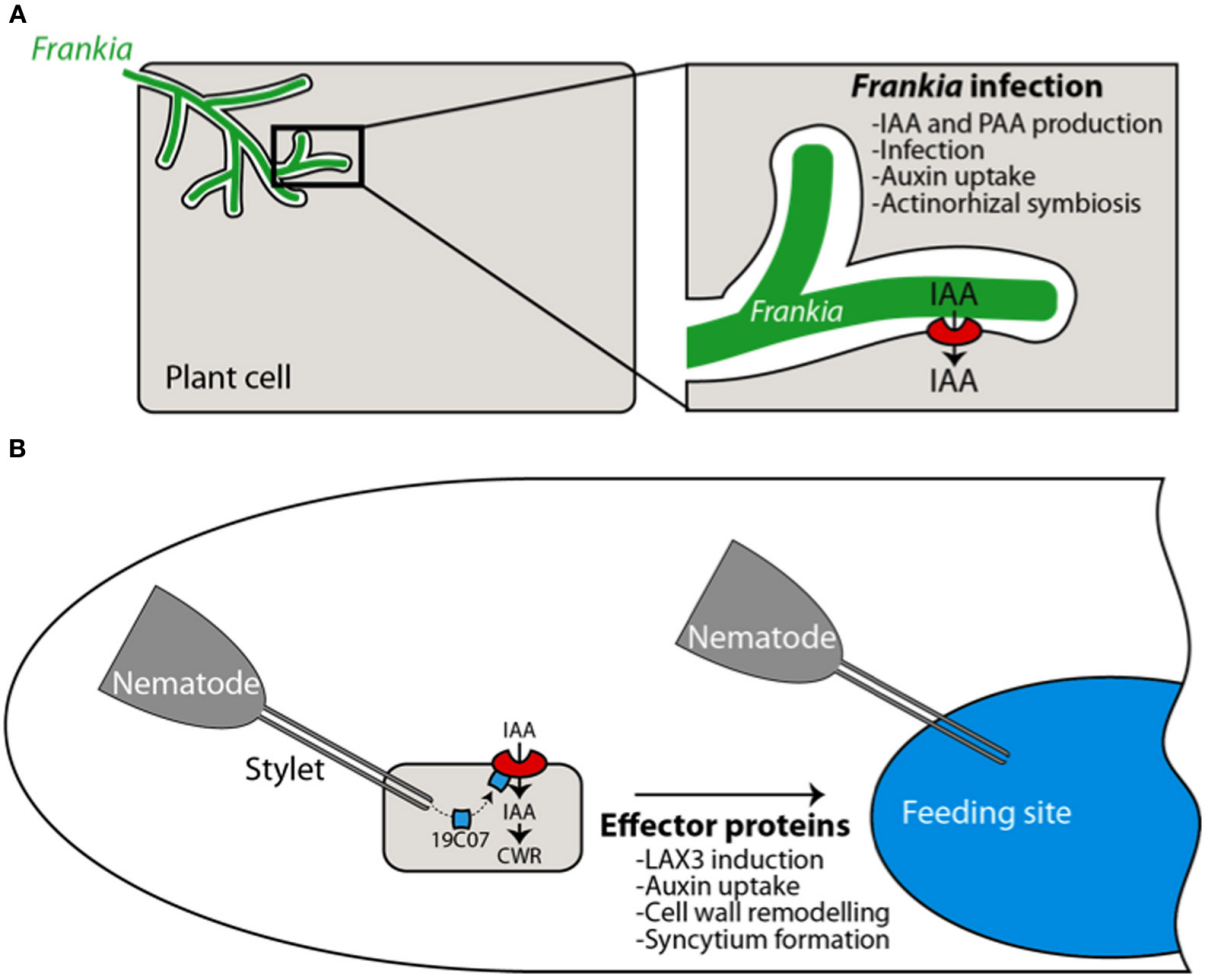
**Auxin influx transporters are involved in biotic interactions**. During the actinorhizal symbiosis, Frankia infects the plant cell and triggers the expression of *CgAUX1*, resulting in auxin (IAA) uptake by the plant. Auxin is presumably synthesized by the actinobacteria (Péret et al., 2007) **(A)**. During cyst nematode infection, effector proteins are released in the plant cell. The 19C07 protein has been shown to directly interact with LAX3. High expression levels of LAX3 in the feeding site and adjacent cells participates in the incorporation of these cells in the feeding sites by promoting cell wall remodeling (CWR) (Lee et al., 2011) **(B)**.

In actinorhizal plant *Casuarina glauca*, a symbiotic interaction with soil actinobacteria from the *Frankia* species leads to infection of the host plant cell and subsequent development of a new organ “the actinorhizal nodule” the site of bacterial nitrogen fixation. Nodule formation in *Casuarina glauca*, can be severely impaired by treatment with auxin influx inhibitor 2-NOA suggesting that auxin influx activity is associated with nodule formation (Péret et al., 2007). This is further supported by molecular studies that show that a homolog of *Arabidopsis AUX1* “Cg*AUX1*” is expressed in all the infected cells, underlining its importance in the infection process (Figure 5A).

Auxin transport has also been implicated in plant pathogen interactions. The cyst nematode is a sedentary endoparasite of plant roots that penetrate the root and migrate toward a cell located near the vasculature to initiate feeding. The nematode then secretes effector proteins in the host cell, leading to genetic reprogramming into a feeding site called a syncytium (Davis et al., 2008). One of these effector proteins (19C07) identified in *Heterodera schachtii* was found to interact with *Arabidopsis* LAX3 auxin transporter in a yeast two-hybrid assay (Lee et al., 2011). The auxin transporter is strongly expressed in the syncytium, together with the auxin inducible cell wall related gene polygalacturonase that is likely to be involved in cell wall loosening. Auxin accumulation in the cells near the syncytium and subsequent cell wall modification would prime the cells for incorporation into the syncytium (Figure 5B). This suggests that the nematode manipulates auxin flow to promote formation of its own feeding site. This is supported by the fact that nematode infectivity is reduced in the *aux1 lax3* double mutant (Lee et al., 2011).

Among the myriad of biotic interactions—both pathogenic and symbiotic, it can be expected that auxin import is involved in a vast number of mechanisms underlying plant interactions with other organisms. A comprehensive study of *AUX-LAX* genes expression during these interactions associated with their functional role both in the model plant *Arabidopsis* and other non-model organisms would greatly improve our understanding of these mechanisms.

## Modeling auxin transport

Modeling studies have provided greater insight into the role of auxin transport in auxin related developmental programmes (Swarup et al., 2005; Kramer and Bennett, 2006; Kramer, 2008; Laskowski et al., 2008; Jones et al., 2009; Prusinkiewicz et al., 2009; Mironova et al., 2010; Szymanowska-Pułka and Nakielski, 2010; Vernoux et al., 2011; Bridge et al., 2012). Modeling auxin fluxes help us to understand how these fluxes are established and maintained, as well as their effect on growth and development. For example, recently, a computational approach studied the dynamics of auxin transport by taking into account pH modifications (Steinacher et al., 2012). The model predicts that auxin-induced acidification of cell wall compartments increases the rate of both auxin influx and efflux. This study also emphasizes the role of proton fluxes, an aspect of the auxin transport machinery that has been poorly studied. Modeling studies have already provided unparalleled insight into the role of AUX1 in establishing and maintaining auxin gradients during root gravitropism (Swarup et al., 2005) and root hair development (Jones et al., 2009). Modeling studies also suggest that AUX1-dependent transport in the root epidermis is necessary for gravitropic response but not for LR initiation. LR formation occurs preferentially at the convex side of roots. Lucas et al. (2008a,b) showed that the LR formation can also be induced by forcing root gravitropic response and proposed a mechanistic model based on an auxin budget system to describe auxin consumption by LR initiation and gravitropic response. Thus, modeling approaches are providing greater insight into the dynamics of auxin distribution and are likely to be at the forefront in the prediction of testable hypothesis of how auxin fluxes control plant development.

## Conclusion and perspectives

In the last decade, genetic and cell biology approaches have resulted in greater understanding of molecular basis of cellular auxin transport. Auxin concentration in plant is affected by either changes in its metabolism or transport, both of which are altered to control plant development (Petrásek and Friml, 2009; Vanneste and Friml, 2009). Auxin influx carriers play a key role in regulating auxin homeostasis. It has been shown that their targeting is cell type specific (Péret et al., 2012) and this adds another level of regulation at tissue level. Identification of proteins that regulate their targeting will provide further insight into their localization and how this affects auxin distribution. Modeling studies have also been crucial in highlighting the role of auxin influx carriers in establishing and maintaining auxin gradients during root gravitropism (Swarup et al., 2005) and root hair development (Jones et al., 2009). Further refinement of the models taking into account all auxin transporters including AUX/LAX, PIN, PGP, and PILS promise to provide further understanding of the role of auxin transporters in auxin distribution.

### Conflict of interest statement

The authors declare that the research was conducted in the absence of any commercial or financial relationships that could be construed as a potential conflict of interest.
